# Kidnapped But Not Kids: A Case Series of Three Octogenarian Hostages Held in Captivity by Hamas

**DOI:** 10.5041/RMMJ.10534

**Published:** 2024-10-28

**Authors:** A. Mark Clarfield, Hagai Levine

**Affiliations:** 1 Faculty of Health Sciences, Ben-Gurion University of the Negev, Be’er Sheva, Israel; 2 Braun School of Public Health and Community Medicine, Hadassah Medical Center, Faculty of Medicine, Hebrew University of Jerusalem, Jerusalem, Israel

**Keywords:** elder abuse, Gaza, hostages, Israel, older persons, resilience, terrorism

## Abstract

On 7 October, 2023 Israel was attacked over the Gaza border by Hamas terrorists. Mostly civilians, approximately 1,200 people were killed, with an additional 251 taken hostage (in addition to 4 abducted before October 7 for a total of 255 hostages), many of whom have since died. Of the total abducted, 13% were older than 65, a third of whom were octogenarians. Brief case histories of three abductees over 80 years of age are presented: two released and one still in captivity. The extreme “pre-morbid” vulnerability of these older hostages is described along with the additional data on their clinical situation and the extreme stresses to which they are being subjected. The situation described constitutes one of the most severe examples of elder abuse documented in the modern era.

## INTRODUCTION

“There is no greater mitzvah [religious commandment] than redeeming captives.”

Maimonides (Moses ben Maimon, rabbi and physician, 1138–1204)

As is now well known, on October 7, 2023, nearly 1,200 Israelis and non-Israelis, mostly civilians, were murdered on the Gaza border. People of all ages were taken hostage, including many older persons, one of whom was a 91-year-old Holocaust survivor gunned down in front of his home.^1^ Evidence indicates that extensive sexual violence occurred during the initial attack and may still be ongoing, as acknowledged by the United Nations^2^ and reported in detail in the lay press.^3^

Following this attack, a fierce war has broken out (ongoing at the time of publication), resulting in the displacement of many hundreds of thousands on both sides of the border, within Gaza and including both southern and northern Israel, and the deaths of tens of thousands of Gazans and many hundreds of Israelis—a huge and ongoing tragedy. This conflict has now spread beyond Hamas’s attack on Israel to encompass assaults against Israeli civilians by Hezbollah from Lebanon in the north, Houthis from Yemen in the south, and Iran in the east.

On the Israeli side, in addition to those murdered on October 7, 251 people, mostly civilians, have been kidnapped, with fewer than half (117) having returned to date: 109 through an exchange and 8 rescued by the Israeli army.^4^ To date, 37 bodies have been retrieved,^5^ including 6 murdered more recently as Israeli soldiers were closing in. Of the total hostages, 33 (13%) are older persons (>65 years), of whom a third (12) are >80 years old.

The condition of some of the released child hostages was recently documented, indicating the terrible conditions in which all of the hostages are likely being held.^6^ However, to the best of our knowledge, there is very little in the medical literature discussing the health effects of long-term abduction on older persons. We found only one paper relating to the health of released captives that included older persons, but that paper did not specifically deal with the health effects on those over 65 years of age.^7^ For its part, Human Rights Watch has produced a useful report on the vulnerability of older persons caught up in armed conflict. However, again, they did not specifically relate to the health status of hostages—either still in captivity or released.^8^

To date there are still 101 hostages in Gaza, most of whom are most likely being held underground (including the 4 taken before October 7). These captives include children, men, and women of all ages, including disabled and older persons, the last-mentioned being the subject of this paper. Of the 33 older hostages, just over half (17) remain in captivity, almost two-thirds (10) of whom are reliably considered to have been murdered, their bodies remaining in captivity. The ambiguous loss and the lack of certainty and proper burial are causing great anguish to their families and to the public.

The eldest hostage is Shlomo Manzur, aged 86 years. His life story includes a terrible irony: fluent in Arabic, as a toddler in Baghdad, Iraq he bore witness to atrocities and survived the infamous 1941 Farhud Pogrom, an event in which 180 Jews were killed and more than 1,000 injured; this outrage is part of the Holocaust that occurred in Arab countries.^9^ Today, in his old age, he has been kidnapped, and it is unknown if he is dead or alive.

While the hostages are primarily Israeli Jews, among them are also Israeli Arabs as well as people of various other religions and nationalities. Many still remain unaccounted for, and at least half are most likely dead due to the dire combination of untreated trauma, lack of medication for chronic diseases, and the poor conditions (i.e. sanitation, ventilation) and diet of those who continue to be held underground—not to mention active torture, abuse, and murder.^10^

Understandably, surrounding this conflict, much has been published about victims of the war aboveground and the dire effects on their health. However, it is surprising that no reports could be found in the medical literature describing the clinical situation of, or active demands for the release of, those held underground in Hamas’s tunnel prisons. All 251 kidnapped individuals are victims of war crimes, as defined by international law and outlined by the International Committee of the Red Cross (ICRC).^11^ Yet, calls for their release seem pro forma, muted, or even worse—absent. For example, a United Nations press release focusing only on the state of the Gazan people is all too typical.^12^

## THREE CASE HISTORIES

At least with respect to the older captives, this report aims to offer some preliminary data provided by families who have approved them for publication (see Table 1). The list provided therein is not exhaustive. As might be expected from their age, many of the older hostages suffer from a variety of ailments common in advanced age, such as diabetes, hypertension, and cardiovascular, respiratory, and renal diseases, as well as mental health problems. For proper control of their conditions, such people need a steady supply of their medications and ongoing medical attention.

**Table 1:** Known Medical Status for a Sample of Male Hostages (>65 years).*

Name	Age (Years)	Primary Medical Conditions	Current Status
Keith Samuel Siegel	65	Hypertension, anxiety, migraine, fibromyalgia	Unknown†
Alexander Dancyg	75	Severe myocardial infarction several years earlier, chronic renal failure; required CPAP for sleep	Did not survive captivity
Avraham Munder	79	Parkinson’s disease, diabetes mellitus, hypertension, vision deficits	Did not survive captivity
Chaim Peri	79	Cancer and cardiovascular disease (on blood thinners when taken); video broadcast in December 2023 by his captors revealed that he had lost a significant percentage of his body weight and muscle tissue^13^	Did not survive captivity
Gadi Mozes	79	Digestive and cardiovascular system disorders	Unknown†
Yoram Metzger	80	Diabetes mellitus and other medical problems requiring essential medications; fractured hip several months before the abduction; testimonies of survivors state he was in constant pain while in captivity	Did not survive captivity
Oded Lifshitz	83	Respiratory and cardiovascular system disorders	Unknown†

* Data obtained from Levine et al.^14^

† As of October 10, 2024.

This descriptive paper presents three brief case reports of three of the older hostages. Two have been released, and the third remains in captivity—his fate unaccounted for. Data sources include medical histories and physical examinations undertaken during home visits with the two released hostages, together with an examination of the medical records of all three hostages. The released hostages gave written permission for the use and publication of their personal data (including identifying data); the immediate family of the hostage still in captivity gave permission for the same.

### Case 1: 85-year-old Woman

Born in Israel, Yocheved Lifshitz was 85 when she was kidnapped on October 7, 2023 along with her husband Oded from their home on Kibbutz Nir Oz, a collective village just a few kilometers from the Gaza–Israel border. She has worked as a photographer and taught both photography and physical education. Approximately a quarter of the 400 members of her collective village were killed or abducted. The couple had lived there for many years and worked hard to develop good relations with their Gazan neighbors.^15^

Mrs Lifshitz had long suffered from several conditions including diabetes, back pain due to herniated disks, renal failure, arrythmia (requiring a pacemaker), cardiac valvular disease, and pulmonary hypertension. Controlling this panoply of pathologies necessitated 17 medications (including beta-blockers, proton-pump inhibitors, anti-coagulants, empagliflozin+metformin, levothyroxine, etc.). Although she did receive some medications from her captors, Mrs Lifshitz was not offered all her required drugs, and it was doubtful that she had been administered the correct medications in their proper doses.

It is clear what would have happened had she been denied any of these medications for a protracted period (as described in our second report below). For reasons that are unclear to this day (most probably related to her captors’ fear that she would infect them after she began to suffer from diarrhea), Mrs Lifshitz was released after “only” two weeks in captivity.

### Case 2: 83-year-old Man

Mr Oded Lifshitz, Yocheved’s husband, was 83 years old on October 7, 2023. Also born in Israel, he is a renowned journalist and one of the founders of Kibbutz Nir Oz. For many years, Mr Lifshitz drove Palestinians from the Gaza border to hospitals in Israel for medical care, as a volunteer with the Road to Recovery Foundation.^16^

His medical condition is even more complex than that of his wife and includes significant respiratory and cardiovascular disease. Among other medications, he was taking Trelegy Ellipta (fluticasone/ umeclidinium/vilanterol), an ACE inhibitor, acetylsalicylic acid, carbocysteine, a statin, iron, and vitamin D. When last seen by his wife, he was lying unconscious outside of their looted house during the violent abduction. Although Mrs Lifshitz had considered him dead, it was only later, when another released hostage reported seeing Mr Lifshitz alive in the tunnels but in very poor shape, that she realized he had not been killed—at least not initially. However, it is unlikely Mr Lifshitz could survive for long without a steady supply of most of his medications and proper medical follow-up.

### Case 3: 85-year-old Woman

Mrs Elma Avraham was nearly 85 when taken hostage. Before being abducted, she was living independently in her home in Kibbutz Nahal Oz, in a stable medical condition, despite suffering from several illnesses. Mrs Avraham’s medical details, her rescue, and the subsequent dramatic life-saving therapy she received in an Israeli hospital are the subject of a forthcoming paper.^17^ Released in critical condition after 51 days of captivity, Mrs Avraham has a medical history that includes hypothyroidism, cutaneous vasculitis, ischemic heart disease, and a transcatheter aortic valve implantation five years before her capture. Medications prescribed include levothyroxine, aspirin, ramipril, lercanidipine, rosuvastatin, and amitriptyline. Mrs Avraham did not receive the medications she needed from her captors but was able to grab some of her pills when abducted. This resourcefulness likely saved her life.

**Figure 1:**
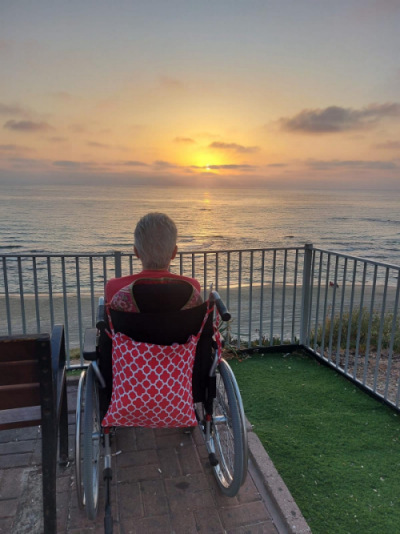
Mrs. Elma Avraham, 7 Months After Her Release From Captivity (Sunset View of the Eastern Mediterranean Sea Near Her New Home). Credit: The Family.

Among those on the Israeli side awaiting her return, there was a strong clinical suspicion that Mrs Avraham would be severely hypothyroid upon release. Upon her return as part of a prisoner swap, she was indeed found to be in myxedema coma: rectal temperature 28.6°C, pulse 40 beats per minute, atrial fibrillation, blood pressure 68/40 mmHg. This was a dire clinical picture rarely observed in modern practice. After 8 days in an intensive care unit, she was transferred to the hospital’s geriatric ward for rehabilitation. After several months, Mrs Avraham was discharged from hospital to a senior center (Figure 1). She could not be released elsewhere since, as was the situation for Mrs Lifshitz and hundreds of others, there was no “home” to return to.

**Figure 2:**
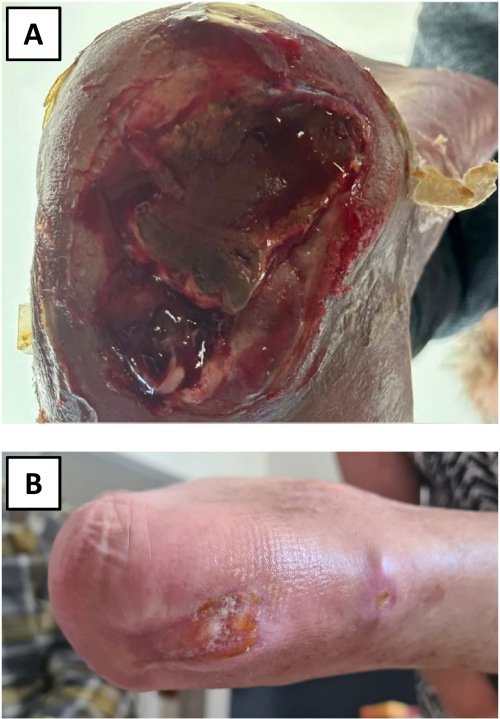
Mrs Elma Avraham; Pressure Sore. Partially healed pressure sore, right calcaneus, which developed following abuse in captivity. **A:** January 31, 2024. **B:** June 6, 2024.

When queried, Mrs Avraham was uncertain where she will spend her remaining years. During a recent visit with her she was calm, albeit subdued. Although ready to talk about her terrible ordeal, the authors were very careful not to press too hard when the questions seemed intrusive. Today, Mrs Avraham’s activities of daily living are markedly reduced. Among other physical challenges she suffers from an unhealed pressure sore acquired in captivity (Figure 2), is dependent on a wheelchair for mobility, and requires 24-hour personal help.

## DISCUSSION

During recent interviews with the authors, both Mrs Lifshitz and Mrs Avraham have expressed a clear inability to recover completely until all the remaining hostages return home—the living for rehabilitation and the dead for burial. The authors have also interviewed the family members of other hostages: most have expressed this same sentiment.

For the hostages still in captivity, many of whom have similar medical needs to those reported upon herein, there is no evidence that they have received *any* of their medications, even after a well-publicized effort by HL and others was made to provide them.^18^,^19^ Furthermore, there is disturbing evidence that the remaining hostages did not, in the end, receive any of these promised medications.^20^

Although exact information about the health status of older hostages and their physical condition is limited, as alluded to above, a disturbing report detailing the experience of released child hostages was recently published.^6^ This account offers clear evidence of extremely difficult physical conditions: physical and psychological abuse, insufficient food and water, as well as inadequate nutrition. As pointed out for the child hostages, the most common findings upon their return included “significant weight loss, psychological trauma, complications of poor hygiene and complications of recent shrapnel injuries. Microbiology tests were positive for multiple gastrointestinal pathogens. Serologic screening tests were positive for various infectious diseases.”^6^(p One can only surmise that older persons are suffering in the same or, given their lack of reserve, an even more severe manner.

As most medical readers of this journal will know, older persons are not as robust as their younger counterparts. Especially after age 80, sarcopenia, deficits in vision and hearing, and other “normal” degenerative changes are common.^21^ In addition to the normal vicissitudes of aging, even reasonably functional older persons often suffer from various diseases and disabilities. In such people, these developments result in a loss of clinical reserve, even in those who do not exhibit any particular comorbidity. In addition to normal aging changes, there is a frequent superimposition of age-related diseases (e.g. diabetes, cardiac and neurological disorders, orthopedic conditions, and cognitive decline). For proper treatment, all patients with such conditions require a steady supply of relevant medications and ongoing supervision. Discontinuing these supports abruptly, as has been the case for the many hostages requiring medication, is a recipe for disaster.

And yet, it is unwise to sell older people short—even those who have been through so much. Older people have accomplished one major feat: they have all survived into old age, and many, despite everything, have said “Yes to Life.”^22^ These older people shine a new light on the word “resilience.”

The three octogenarian hostages described herein all exhibit various manifestations of these more generalized phenomena. Each of them made it into old age, having lived for decades on kibbutzim [agricultural collectives] very close to the Gaza border, two having survived abduction to Gaza.

For those older captives who have survived now, and especially for the more than 100,000 Holocaust survivors (almost all over age 85) who reside in Israel, these abductions and the ongoing captivity of so many causes them to question whether the punctuation mark in “Never Again!” should be replaced by a question mark.^23^

What have we learned from our examination of these older persons? As a young physician almost half a century ago, one of us (AMC) enjoyed the privilege of caring for veterans of the First World War. To him it was indeed an honor to be able to pay one’s “last respects.” The second author (HL) was inspired by knowing his great-grandfather who had himself been a physician during this same conflict. More recently, both authors have had the privilege of looking after the last remaining survivors of the Holocaust as they, too, are reaching the end of their long and traumatized lives.

But these hostage cases in Gaza are different. Their captivity is ongoing and continues in real time, and these vulnerable people are all victims of severe elder abuse—to the best of our knowledge the likes of which have not previously been documented in the literature.

What has been done, outside of Israel, to advocate for the medical needs of the remaining hostages—old and young? Unfortunately, very little. For example, the International Committee of Red Cross (ICRC) mandate to provide humanitarian protection and assistance to victims of armed conflict, and to ensure adherence with international humanitarian law, has been less than proactive. In the view of many observers, the ICRC has not utilized all of their available tools to gain access to hostages and provide them with the necessary aid.

When queried by one of the authors (HL) in his lead health role in the Hostages and Missing— Families Forum, a member of the ICRC leadership responded that members of this organization are meant to be “neutral” and thus could do no more. However, in the view of many outside observers, respecting such a position hardly precludes this important organization from fulfilling its mandate by making every possible effort to provide protection to those affected by armed conflicts. Furthermore, although the ICRC has indeed asked Hamas for information about the remaining hostages, none has been forthcoming. However, a less than robust “what else can we do?” from this important NGO has been a source of disappointment to the hostages’ families themselves and to those advocating for their release. One of us (HL) recalls witnessing a disturbing lack of empathy expressed when senior ICRC representatives were asked by Mrs Avraham’s son to ensure that she would receive all her medications.^24^

Indeed, it is legitimate to ask why the organization is not taking a more proactive stance. Doing so would avoid finding themselves in the future position of having to make another belated apology for inaction today, as they did in the past with regard to their shameful and regretted silence during the Holocaust.^25^

For their part, the World Health Organization (WHO), other United Nations bodies, and many humanitarian non-governmental organizations, while quick to condemn Israel for injuries to Gazans, appear to offer limited or only lip service in support of an immediate release of all those illegally abducted by Hamas. The response of most medical professional bodies has been equally disappointing although the occasional exception is noted^26^ and appreciated by hostages, young and old, as well as those advocating for them.

Understandably, much has been written about the suffering of the Palestinians above ground and how Gazan civilians are cynically exploited as human shields by Hamas.^27^ As humans and humanitarian workers, we can only share in this anguish. However, so little has appeared in the medical literature or in professional declarations calling for the release of the sick and abused Israeli hostages under Gaza’s surface—old or young—that it forces one to question the motives of some of those advocating so insistently for Gazan civilians whilst ignoring the plight of Israeli victims.

## CONCLUSION

What has been learned from our contact with these three older victims of terrorism? As alluded to above, as physicians, given that older persons are by definition survivors, perhaps we should not be surprised (although certainly heartened) by the observation that some can endure even the most trying conditions. These old people are exemplars. The greater tragedy, of course, is that in their old age, instead of being helped to fulfill their potential and enjoy a well-deserved rest, they have been challenged so barbarously.

Recently, a Commission on Nazi Medicine and the Holocaust tabled its report.^28^ Staunchly, in an effort to offer a contemporary lesson from a dark history, they called for the fostering of history-informed, morally courageous health professionals who will speak up when necessary. Furthermore, the Commission asserts the importance of holding the perpetrators accountable.

However, despite such timely calls, most of the medical world remains silent. Whether any of the older hostages still in captivity live to tell the tale or not remains to be seen. Hopefully, the publication of case reports such as those described herein will spur more advocacy.

## Abbreviations

ICRC: International Committee of the Red Cross.
